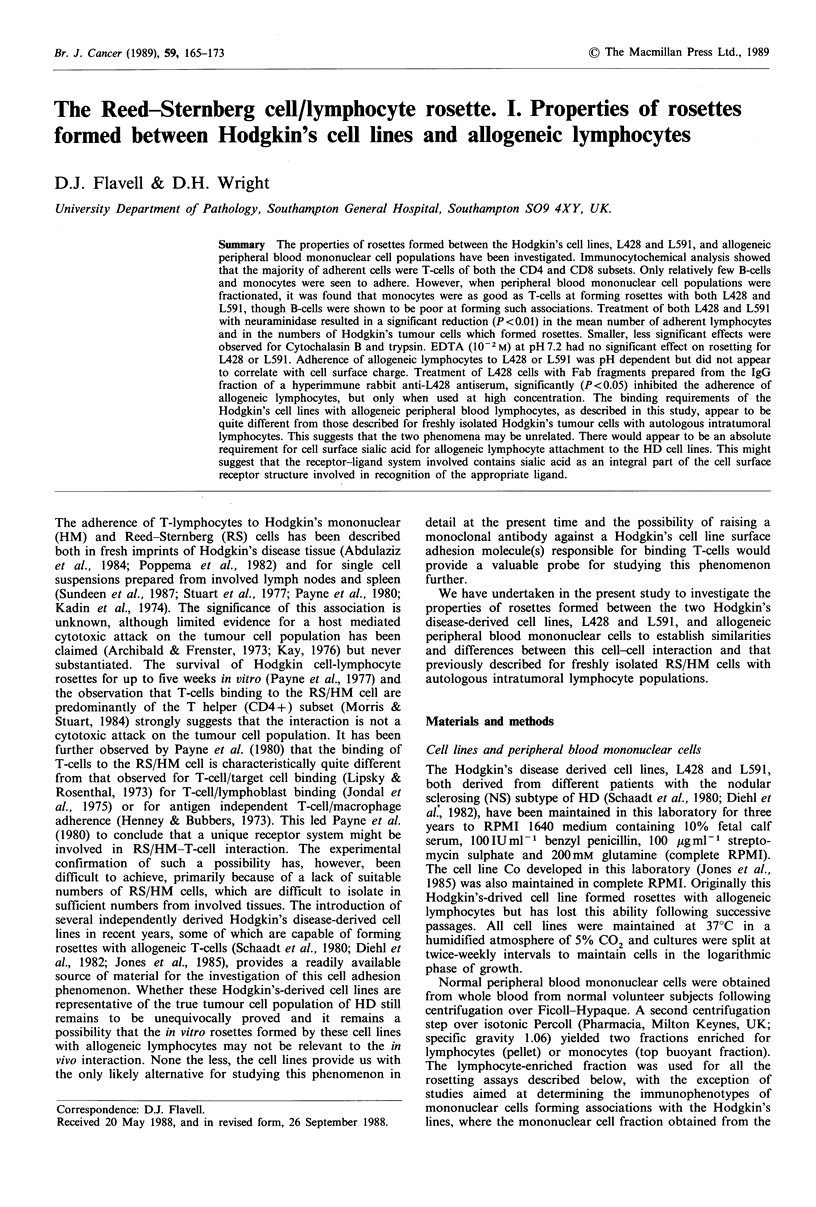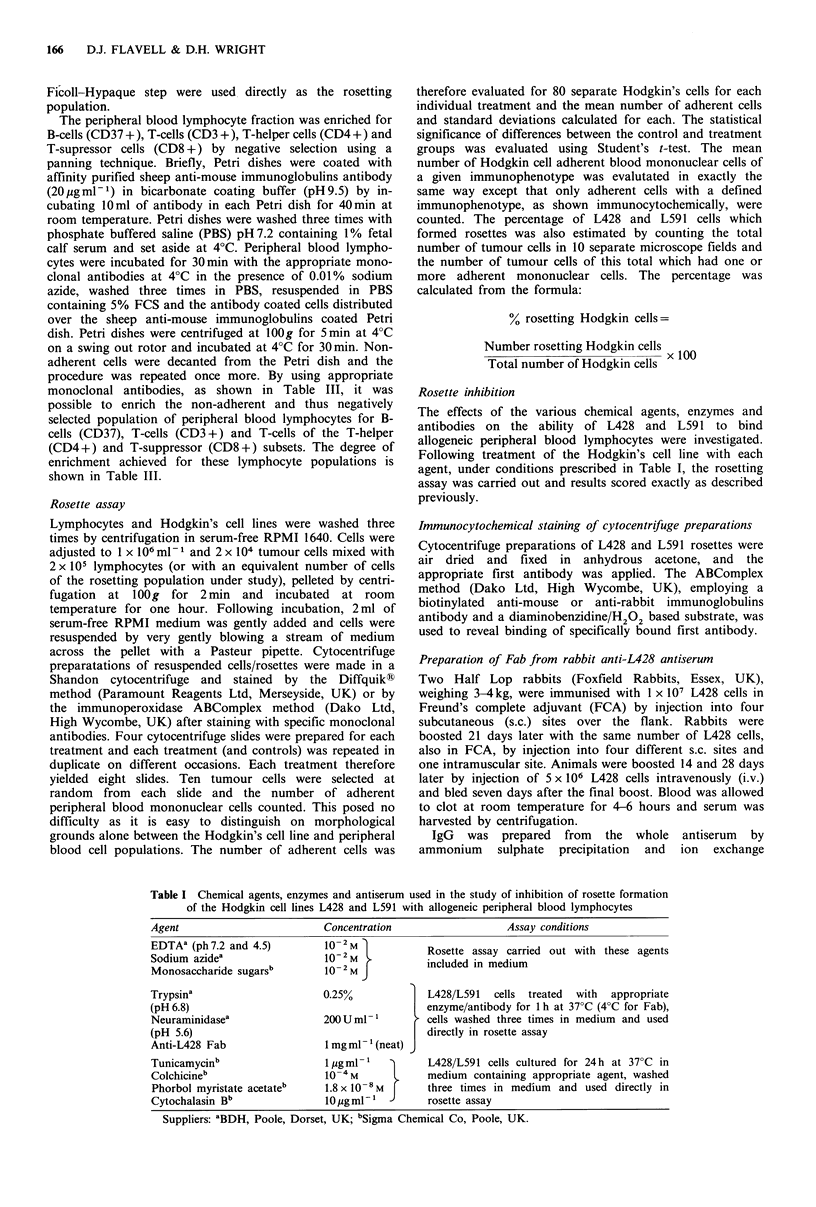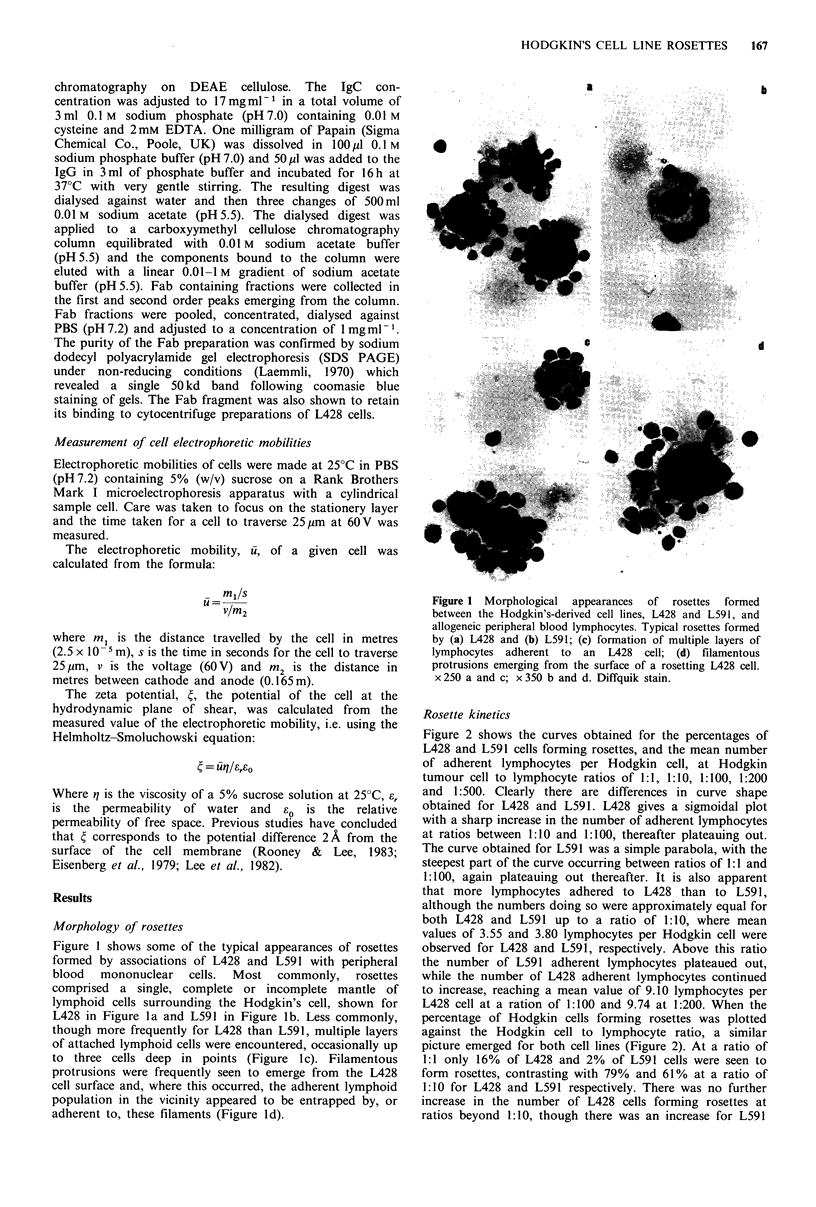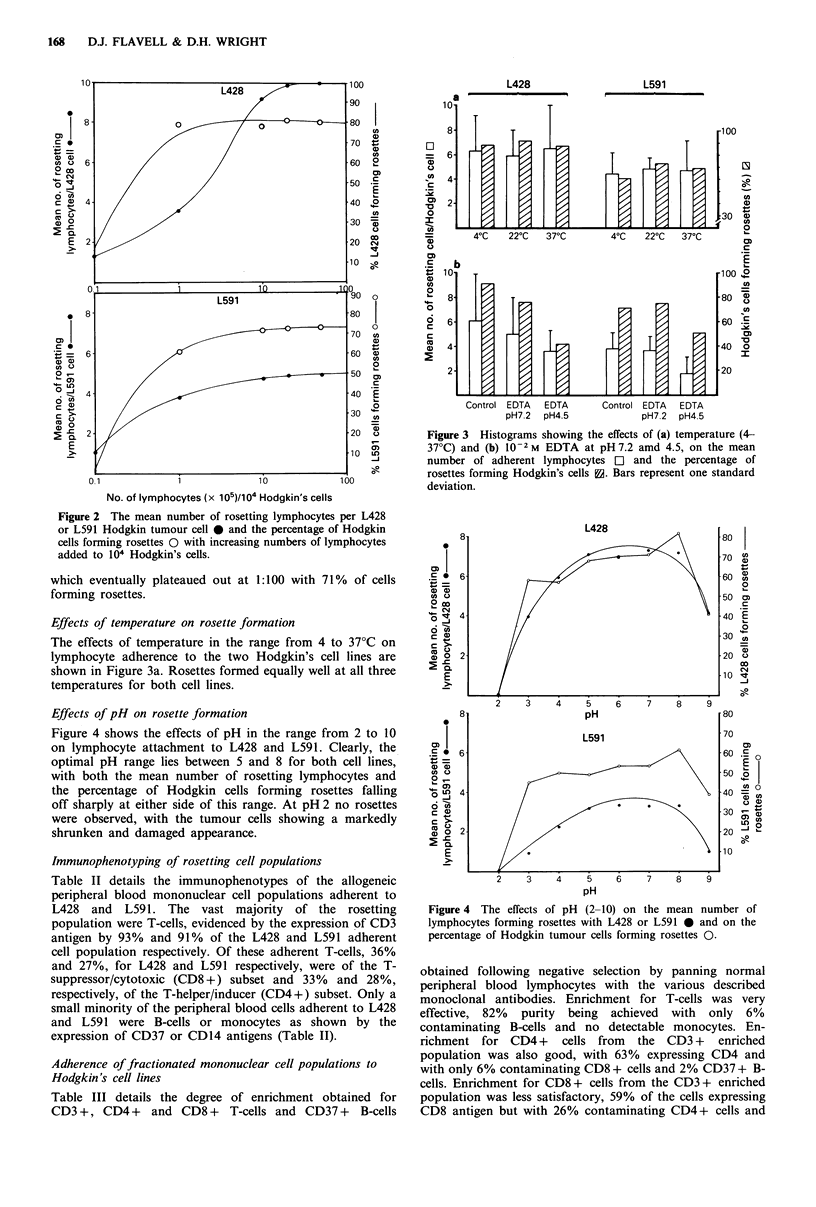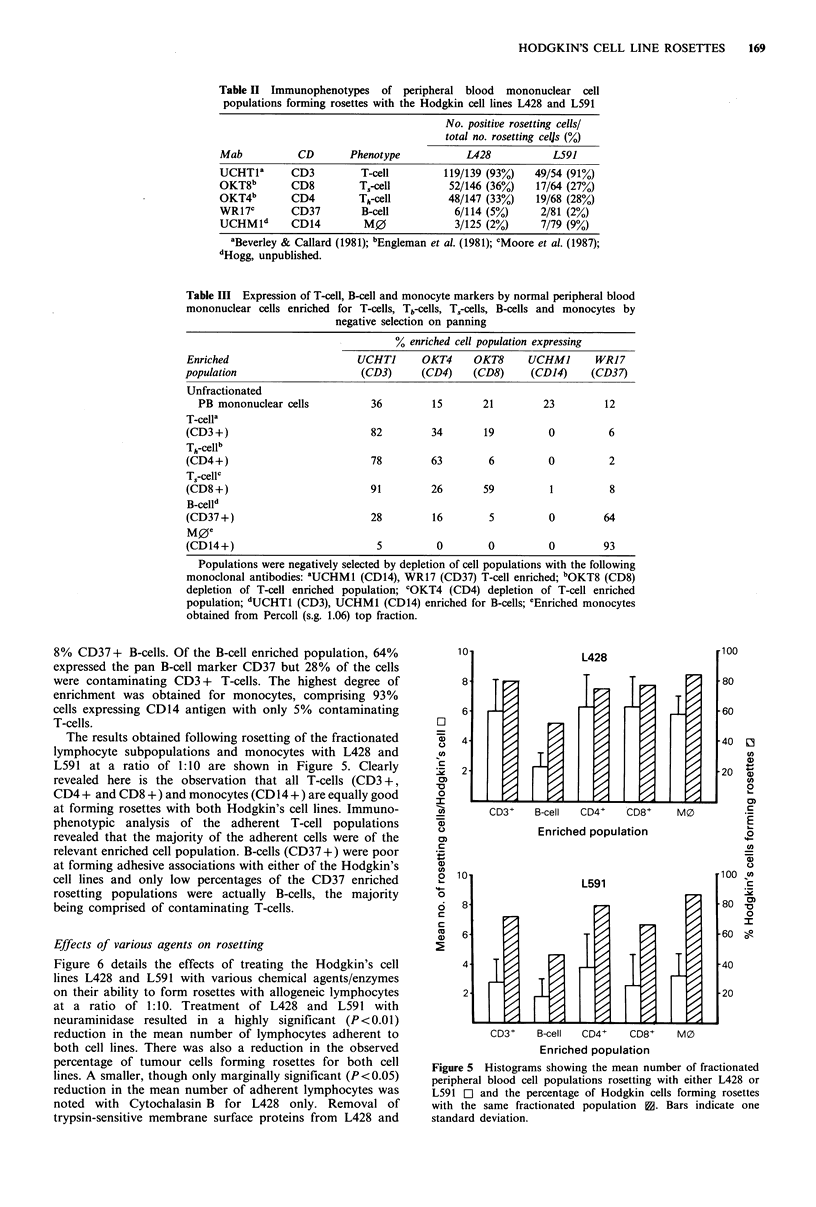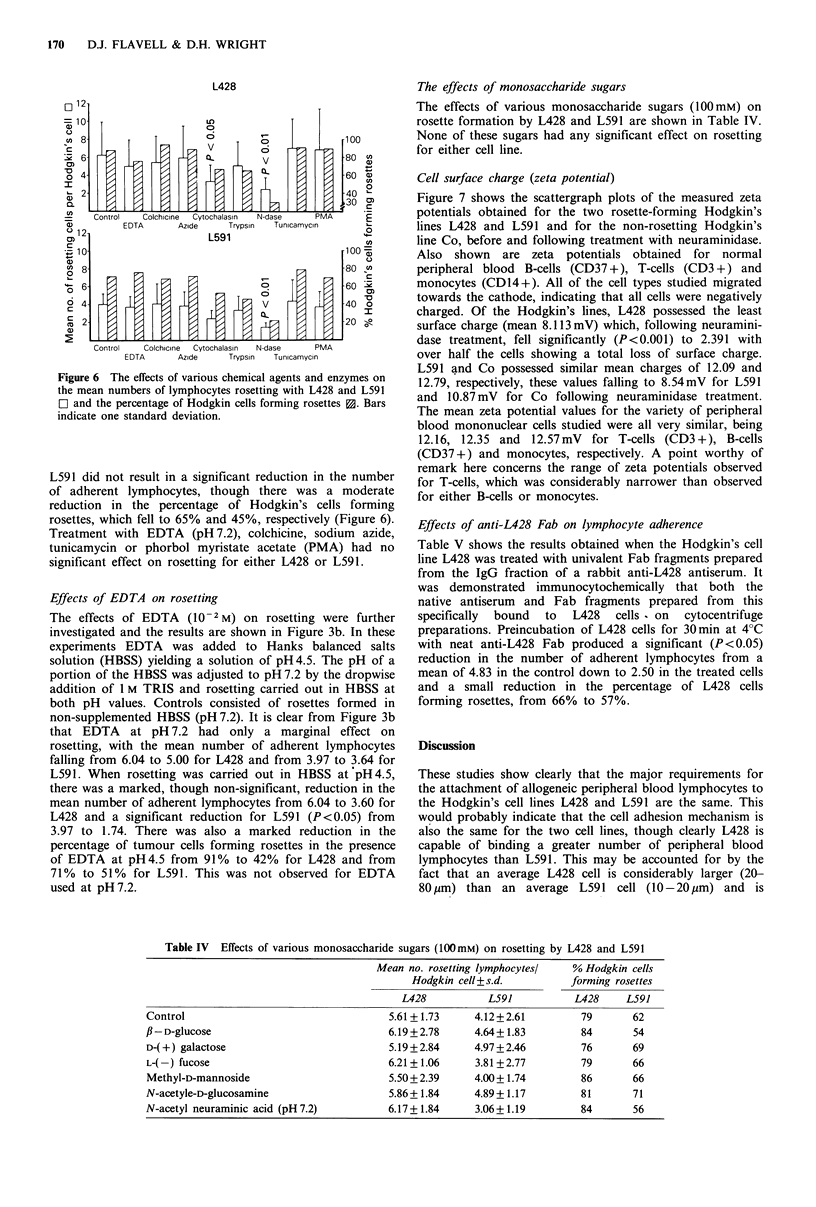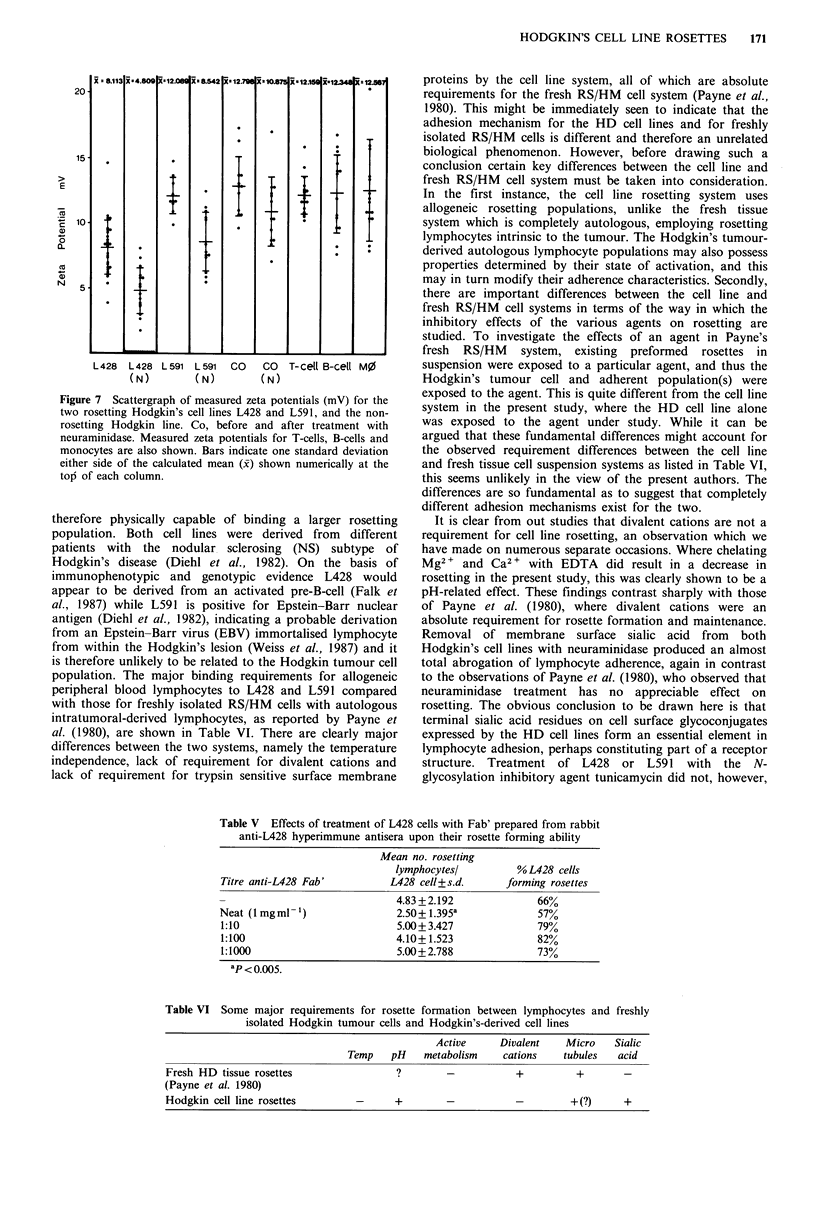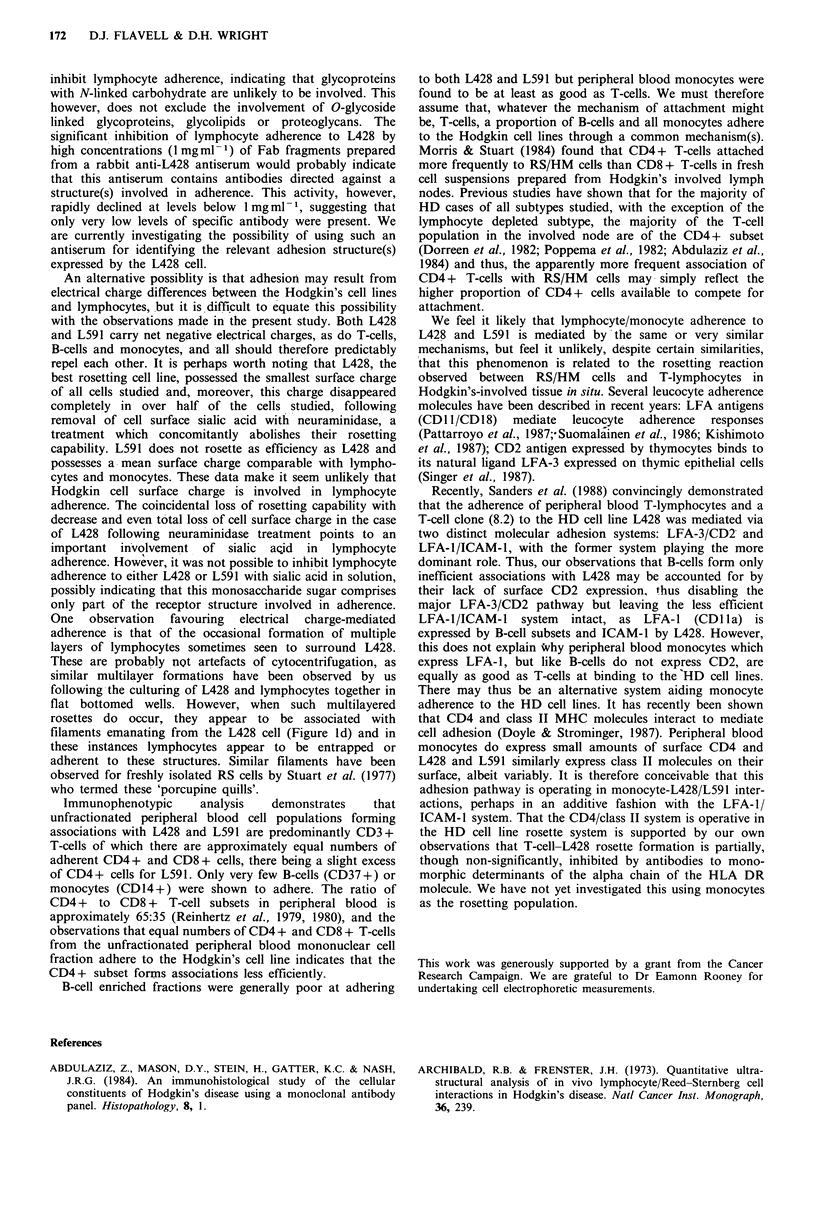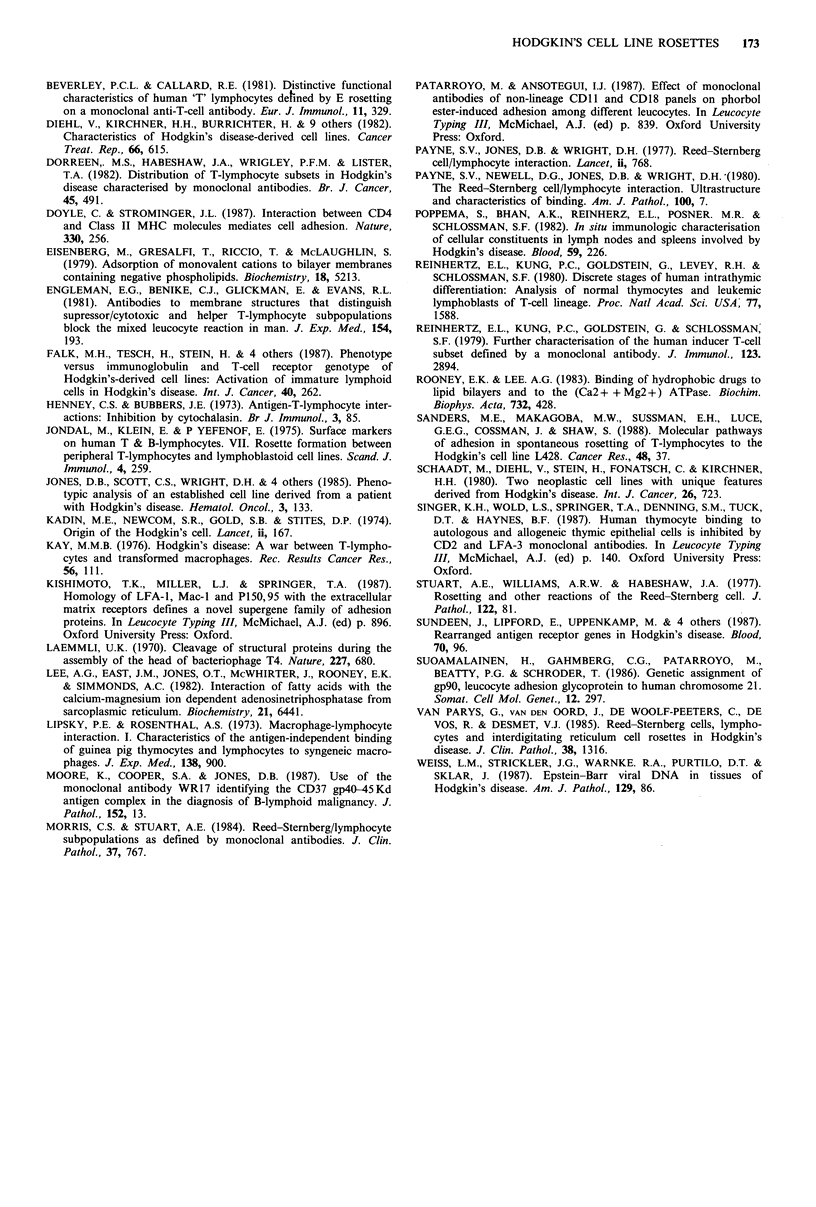# The Reed-Sternberg cell/lymphocyte rosette. I. Properties of rosettes formed between Hodgkin's cell lines and allogeneic lymphocytes.

**DOI:** 10.1038/bjc.1989.35

**Published:** 1989-02

**Authors:** D. J. Flavell, D. H. Wright

**Affiliations:** University Department of Pathology, Southampton General Hospital, UK.

## Abstract

**Images:**


Br. J. Cancer (1989), 59, 165–173 © The Macmillan Press Ltd., 1989

### The Reed–Sternberg cell/lymphocyte rosette. I. Properties of rosettes formed between Hodgkin's cell lines and allogeneic lymphocytes

D.J. Flavell & D.H. Wright

*University Department of Pathology, Southampton General Hospital, Southampton SO9 4XY, UK.*

**Summary** The properties of rosettes formed between the Hodgkin's cell lines, L428 and L591, and allogeneic peripheral blood mononuclear cell populations have been investigated. Immunocytochemical analysis showed that the majority of adherent cells were T-cells of both the CD4 and CD8 subsets. Only relatively few B-cells and monocytes were seen to adhere. However, when peripheral blood mononuclear cell populations were fractionated, it was found that monocytes were as good as T-cells at forming rosettes with both L428 and L591, though B-cells were shown to be poor at forming such associations. Treatment of both L428 and L591 with neuraminidase resulted in a significant reduction ($P<0.01$) in the mean number of adherent lymphocytes and in the numbers of Hodgkin's tumour cells which formed rosettes. Smaller, less significant effects were observed for Cytochalasin B and trypsin. EDTA ($10^{-2}$ M) at pH 7.2 had no significant effect on rosetting for L428 or L591. Adherence of allogeneic lymphocytes to L428 or L591 was pH dependent but did not appear to correlate with cell surface charge. Treatment of L428 cells with Fab fragments prepared from the IgG fraction of a hyperimmune rabbit anti-L428 antiserum, significantly ($P<0.05$) inhibited the adherence of allogeneic lymphocytes, but only when used at high concentration. The binding requirements of the Hodgkin's cell lines with allogeneic peripheral blood lymphocytes, as described in this study, appear to be quite different from those described for freshly isolated Hodgkin's tumour cells with autologous intratumoral lymphocytes. This suggests that the two phenomena may be unrelated. There would appear to be an absolute requirement for cell surface sialic acid for allogeneic lymphocyte attachment to the HD cell lines. This might suggest that the receptor–ligand system involved contains sialic acid as an integral part of the cell surface receptor structure involved in recognition of the appropriate ligand.

The adherence of T-lymphocytes to Hodgkin's mononuclear (HM) and Reed–Sternberg (RS) cells has been described both in fresh imprints of Hodgkin's disease tissue (Abdulaziz *et al.*, 1984; Poppema *et al.*, 1982) and for single cell suspensions prepared from involved lymph nodes and spleen (Sundeen *et al.*, 1987; Stuart *et al.*, 1977; Payne *et al.*, 1980; Kadin *et al.*, 1974). The significance of this association is unknown, although limited evidence for a host mediated cytotoxic attack on the tumour cell population has been claimed (Archibald & Frenster, 1973; Kay, 1976) but never substantiated. The survival of Hodgkin cell-lymphocyte rosettes for up to five weeks *in vitro* (Payne *et al.*, 1977) and the observation that T-cells binding to the RS/HM cell are predominantly of the T helper (CD4+) subset (Morris & Stuart, 1984) strongly suggests that the interaction is not a cytotoxic attack on the tumour cell population. It has been further observed by Payne *et al.* (1980) that the binding of T-cells to the RS/HM cell is characteristically quite different from that observed for T-cell/target cell binding (Lipsky & Rosenthal, 1973) for T-cell/lymphoblast binding (Jondal *et al.*, 1975) or for antigen independent T-cell/macrophage adherence (Henney & Bubbers, 1973). This led Payne *et al.* (1980) to conclude that a unique receptor system might be involved in RS/HM–T-cell interaction. The experimental confirmation of such a possibility has, however, been difficult to achieve, primarily because of a lack of suitable numbers of RS/HM cells, which are difficult to isolate in sufficient numbers from involved tissues. The introduction of several independently derived Hodgkin's disease-derived cell lines in recent years, some of which are capable of forming rosettes with allogeneic T-cells (Schaadt *et al.*, 1980; Diehl *et al.*, 1982; Jones *et al.*, 1985), provides a readily available source of material for the investigation of this cell adhesion phenomenon. Whether these Hodgkin's-derived cell lines are representative of the true tumour cell population of HD still remains to be unequivocally proved and it remains a possibility that the *in vitro* rosettes formed by these cell lines with allogeneic lymphocytes may not be relevant to the *in vivo* interaction. None the less, the cell lines provide us with the only likely alternative for studying this phenomenon in detail at the present time and the possibility of raising a monoclonal antibody against a Hodgkin's cell line surface adhesion molecule(s) responsible for binding T-cells would provide a valuable probe for studying this phenomenon further.

We have undertaken in the present study to investigate the properties of rosettes formed between the two Hodgkin's disease-derived cell lines, L428 and L591, and allogeneic peripheral blood mononuclear cells to establish similarities and differences between this cell–cell interaction and that previously described for freshly isolated RS/HM cells with autologous intratumoral lymphocyte populations.

### Materials and methods

### *Cell lines and peripheral blood mononuclear cells*

The Hodgkin's disease derived cell lines, L428 and L591, both derived from different patients with the nodular sclerosing (NS) subtype of HD (Schaadt *et al.*, 1980; Diehl *et al.*, 1982), have been maintained in this laboratory for three years to RPMI 1640 medium containing 10% fetal calf serum, 100 IU ml$^{-1}$ benzyl penicillin, 100 $\mu$g ml$^{-1}$ streptomycin sulphate and 200 mM glutamine (complete RPMI). The cell line Co developed in this laboratory (Jones *et al.*, 1985) was also maintained in complete RPMI. Originally this Hodgkin's-drived cell line formed rosettes with allogeneic lymphocytes but has lost this ability following successive passages. All cell lines were maintained at 37°C in a humidified atmosphere of 5% $CO_2$ and cultures were split at twice-weekly intervals to maintain cells in the logarithmic phase of growth.

Normal peripheral blood mononuclear cells were obtained from whole blood from normal volunteer subjects following centrifugation over Ficoll–Hypaque. A second centrifugation step over isotonic Percoll (Pharmacia, Milton Keynes, UK; specific gravity 1.06) yielded two fractions enriched for lymphocytes (pellet) or monocytes (top buoyant fraction). The lymphocyte-enriched fraction was used for all the rosetting assays described below, with the exception of studies aimed at determining the immunophenotypes of mononuclear cells forming associations with the Hodgkin's lines, where the mononuclear cell fraction obtained from the

Correspondence: D.J. Flavell.
Received 20 May 1988, and in revised form, 26 September 1988.

Ficoll–Hypaque step were used directly as the rosetting population.

The peripheral blood lymphocyte fraction was enriched for B-cells (CD37+), T-cells (CD3+), T-helper cells (CD4+) and T-supressor cells (CD8+) by negative selection using a panning technique. Briefly, Petri dishes were coated with affinity purified sheep anti-mouse immunoglobulins antibody ($20\,\mu g\,ml^{-1}$) in bicarbonate coating buffer (pH 9.5) by incubating 10 ml of antibody in each Petri dish for 40 min at room temperature. Petri dishes were washed three times with phosphate buffered saline (PBS) pH 7.2 containing 1% fetal calf serum and set aside at 4°C. Peripheral blood lymphocytes were incubated for 30 min with the appropriate monoclonal antibodies at 4°C in the presence of 0.01% sodium azide, washed three times in PBS, resuspended in PBS containing 5% FCS and the antibody coated cells distributed over the sheep anti-mouse immunoglobulins coated Petri dish. Petri dishes were centrifuged at 100 *g* for 5 min at 4°C on a swing out rotor and incubated at 4°C for 30 min. Non-adherent cells were decanted from the Petri dish and the procedure was repeated once more. By using appropriate monoclonal antibodies, as shown in Table III, it was possible to enrich the non-adherent and thus negatively selected population of peripheral blood lymphocytes for B-cells (CD37), T-cells (CD3+) and T-cells of the T-helper (CD4+) and T-suppressor (CD8+) subsets. The degree of enrichment achieved for these lymphocyte populations is shown in Table III.

*Rosette assay*

Lymphocytes and Hodgkin's cell lines were washed three times by centrifugation in serum-free RPMI 1640. Cells were adjusted to $1\times10^6\,ml^{-1}$ and $2\times10^4$ tumour cells mixed with $2\times10^5$ lymphocytes (or with an equivalent number of cells of the rosetting population under study), pelleted by centrifugation at 100 *g* for 2 min and incubated at room temperature for one hour. Following incubation, 2 ml of serum-free RPMI medium was gently added and cells were resuspended by very gently blowing a stream of medium across the pellet with a Pasteur pipette. Cytocentrifuge preparatations of resuspended cells/rosettes were made in a Shandon cytocentrifuge and stained by the Diffquik® method (Paramount Reagents Ltd, Merseyside, UK) or by the immunoperoxidase ABComplex method (Dako Ltd, High Wycombe, UK) after staining with specific monoclonal antibodies. Four cytocentrifuge slides were prepared for each treatment and each treatment (and controls) was repeated in duplicate on different occasions. Each treatment therefore yielded eight slides. Ten tumour cells were selected at random from each slide and the number of adherent peripheral blood mononuclear cells counted. This posed no difficulty as it is easy to distinguish on morphological grounds alone between the Hodgkin's cell line and peripheral blood cell populations. The number of adherent cells was therefore evaluated for 80 separate Hodgkin's cells for each individual treatment and the mean number of adherent cells and standard deviations calculated for each. The statistical significance of differences between the control and treatment groups was evaluated using Student's *t*-test. The mean number of Hodgkin cell adherent blood mononuclear cells of a given immunophenotype was evalutated in exactly the same way except that only adherent cells with a defined immunophenotype, as shown immunocytochemically, were counted. The percentage of L428 and L591 cells which formed rosettes was also estimated by counting the total number of tumour cells in 10 separate microscope fields and the number of tumour cells of this total which had one or more adherent mononuclear cells. The percentage was calculated from the formula:

$$\%\ \text{rosetting Hodgkin cells} = \frac{\text{Number rosetting Hodgkin cells}}{\text{Total number of Hodgkin cells}} \times 100$$

*Rosette inhibition*

The effects of the various chemical agents, enzymes and antibodies on the ability of L428 and L591 to bind allogeneic peripheral blood lymphocytes were investigated. Following treatment of the Hodgkin's cell line with each agent, under conditions prescribed in Table I, the rosetting assay was carried out and results scored exactly as described previously.

*Immunocytochemical staining of cytocentrifuge preparations*

Cytocentrifuge preparations of L428 and L591 rosettes were air dried and fixed in anhydrous acetone, and the appropriate first antibody was applied. The ABComplex method (Dako Ltd, High Wycombe, UK), employing a biotinylated anti-mouse or anti-rabbit immunoglobulins antibody and a diaminobenzidine/$H_2O_2$ based substrate, was used to reveal binding of specifically bound first antibody.

*Preparation of Fab from rabbit anti-L428 antiserum*

Two Half Lop rabbits (Foxfield Rabbits, Essex, UK), weighing 3–4 kg, were immunised with $1\times10^7$ L428 cells in Freund's complete adjuvant (FCA) by injection into four subcutaneous (s.c.) sites over the flank. Rabbits were boosted 21 days later with the same number of L428 cells, also in FCA, by injection into four different s.c. sites and one intramuscular site. Animals were boosted 14 and 28 days later by injection of $5\times10^6$ L428 cells intravenously (i.v.) and bled seven days after the final boost. Blood was allowed to clot at room temperature for 4–6 hours and serum was harvested by centrifugation.

IgG was prepared from the whole antiserum by ammonium sulphate precipitation and ion exchange

**Table I** Chemical agents, enzymes and antiserum used in the study of inhibition of rosette formation of the Hodgkin cell lines L428 and L591 with allogeneic peripheral blood lymphocytes

| *Agent* | *Concentration* | *Assay conditions* |
|---|---|---|
| EDTA[a] (ph 7.2 and 4.5) | $10^{-2}$ M | Rosette assay carried out with these agents included in medium |
| Sodium azide[a] | $10^{-2}$ M | |
| Monosaccharide sugars[b] | $10^{-2}$ M | |
| Trypsin[a] (pH 6.8) | 0.25% | L428/L591 cells treated with appropriate enzyme/antibody for 1 h at 37°C (4°C for Fab), cells washed three times in medium and used directly in rosette assay |
| Neuraminidase[a] (pH 5.6) | $200\,U\,ml^{-1}$ | |
| Anti-L428 Fab | $1\,mg\,ml^{-1}$ (neat) | |
| Tunicamycin[b] | $1\,\mu g\,ml^{-1}$ | L428/L591 cells cultured for 24 h at 37°C in medium containing appropriate agent, washed three times in medium and used directly in rosette assay |
| Colchicine[b] | $10^{-4}$ M | |
| Phorbol myristate acetate[b] | $1.8\times10^{-8}$ M | |
| Cytochalasin B[b] | $10\,\mu g\,ml^{-1}$ | |

Suppliers: [a]BDH, Poole, Dorset, UK; [b]Sigma Chemical Co, Poole, UK.

chromatography on DEAE cellulose. The IgC concentration was adjusted to 17 mg ml$^{-1}$ in a total volume of 3 ml 0.1 M sodium phosphate (pH 7.0) containing 0.01 M cysteine and 2 mM EDTA. One milligram of Papain (Sigma Chemical Co., Poole, UK) was dissolved in 100 $\mu$l 0.1 M sodium phosphate buffer (pH 7.0) and 50 $\mu$l was added to the IgG in 3 ml of phosphate buffer and incubated for 16 h at 37°C with very gentle stirring. The resulting digest was dialysed against water and then three changes of 500 ml 0.01 M sodium acetate (pH 5.5). The dialysed digest was applied to a carboxyymethyl cellulose chromatography column equilibrated with 0.01 M sodium acetate buffer (pH 5.5) and the components bound to the column were eluted with a linear 0.01–1 M gradient of sodium acetate buffer (pH 5.5). Fab containing fractions were collected in the first and second order peaks emerging from the column. Fab fractions were pooled, concentrated, dialysed against PBS (pH 7.2) and adjusted to a concentration of 1 mg ml$^{-1}$. The purity of the Fab preparation was confirmed by sodium dodecyl polyacrylamide gel electrophoresis (SDS PAGE) under non-reducing conditions (Laemmli, 1970) which revealed a single 50 kd band following coomasie blue staining of gels. The Fab fragment was also shown to retain its binding to cytocentrifuge preparations of L428 cells.

*Measurement of cell electrophoretic mobilities*

Electrophoretic mobilities of cells were made at 25°C in PBS (pH 7.2) containing 5% (w/v) sucrose on a Rank Brothers Mark I microelectrophoresis apparatus with a cylindrical sample cell. Care was taken to focus on the stationery layer and the time taken for a cell to traverse 25 $\mu$m at 60 V was measured.

The electrophoretic mobility, $\bar{u}$, of a given cell was calculated from the formula:

$$\bar{u} = \frac{m_1/s}{v/m_2}$$

where $m_1$ is the distance travelled by the cell in metres ($2.5 \times 10^{-5}$ m), $s$ is the time in seconds for the cell to traverse 25 $\mu$m, $v$ is the voltage (60 V) and $m_2$ is the distance in metres between cathode and anode (0.165 m).

The zeta potential, $\zeta$, the potential of the cell at the hydrodynamic plane of shear, was calculated from the measured value of the electrophoretic mobility, i.e. using the Helmholtz–Smoluchowski equation:

$$\zeta = \bar{u}\eta/\varepsilon_r\varepsilon_0$$

Where $\eta$ is the viscosity of a 5% sucrose solution at 25°C, $\varepsilon_r$ is the permeability of water and $\varepsilon_0$ is the relative permeability of free space. Previous studies have concluded that $\zeta$ corresponds to the potential difference 2 Å from the surface of the cell membrane (Rooney & Lee, 1983; Eisenberg *et al.*, 1979; Lee *et al.*, 1982).

### Results

*Morphology of rosettes*

Figure 1 shows some of the typical appearances of rosettes formed by associations of L428 and L591 with peripheral blood mononuclear cells. Most commonly, rosettes comprised a single, complete or incomplete mantle of lymphoid cells surrounding the Hodgkin's cell, shown for L428 in Figure 1a and L591 in Figure 1b. Less commonly, though more frequently for L428 than L591, multiple layers of attached lymphoid cells were encountered, occasionally up to three cells deep in points (Figure 1c). Filamentous protrusions were frequently seen to emerge from the L428 cell surface and, where this occurred, the adherent lymphoid population in the vicinity appeared to be entrapped by, or adherent to, these filaments (Figure 1d).

**Figure 1** Morphological appearances of rosettes formed between the Hodgkin's-derived cell lines, L428 and L591, and allogeneic peripheral blood lymphocytes. Typical rosettes formed by (**a**) L428 and (**b**) L591; (**c**) formation of multiple layers of lymphocytes adherent to an L428 cell; (**d**) filamentous protrusions emerging from the surface of a rosetting L428 cell. ×250 a and c; ×350 b and d. Diffquik stain.

*Rosette kinetics*

Figure 2 shows the curves obtained for the percentages of L428 and L591 cells forming rosettes, and the mean number of adherent lymphocytes per Hodgkin cell, at Hodgkin tumour cell to lymphocyte ratios of 1:1, 1:10, 1:100, 1:200 and 1:500. Clearly there are differences in curve shape obtained for L428 and L591. L428 gives a sigmoidal plot with a sharp increase in the number of adherent lymphocytes at ratios between 1:10 and 1:100, thereafter plateauing out. The curve obtained for L591 was a simple parabola, with the steepest part of the curve occurring between ratios of 1:1 and 1:100, again plateauing out thereafter. It is also apparent that more lymphocytes adhered to L428 than to L591, although the numbers doing so were approximately equal for both L428 and L591 up to a ratio of 1:10, where mean values of 3.55 and 3.80 lymphocytes per Hodgkin cell were observed for L428 and L591, respectively. Above this ratio the number of L591 adherent lymphocytes plateaued out, while the number of L428 adherent lymphocytes continued to increase, reaching a mean value of 9.10 lymphocytes per L428 cell at a ration of 1:100 and 9.74 at 1:200. When the percentage of Hodgkin cells forming rosettes was plotted against the Hodgkin cell to lymphocyte ratio, a similar picture emerged for both cell lines (Figure 2). At a ratio of 1:1 only 16% of L428 and 2% of L591 cells were seen to form rosettes, contrasting with 79% and 61% at a ratio of 1:10 for L428 and L591 respectively. There was no further increase in the number of L428 cells forming rosettes at ratios beyond 1:10, though there was an increase for L591

Figure 2 The mean number of rosetting lymphocytes per L428 or L591 Hodgkin tumour cell ● and the percentage of Hodgkin cells forming rosettes ○ with increasing numbers of lymphocytes added to $10^4$ Hodgkin's cells.

which eventually plateaued out at 1:100 with 71% of cells forming rosettes.

*Effects of temperature on rosette formation*

The effects of temperature in the range from 4 to 37°C on lymphocyte adherence to the two Hodgkin's cell lines are shown in Figure 3a. Rosettes formed equally well at all three temperatures for both cell lines.

*Effects of pH on rosette formation*

Figure 4 shows the effects of pH in the range from 2 to 10 on lymphocyte attachment to L428 and L591. Clearly, the optimal pH range lies between 5 and 8 for both cell lines, with both the mean number of rosetting lymphocytes and the percentage of Hodgkin cells forming rosettes falling off sharply at either side of this range. At pH 2 no rosettes were observed, with the tumour cells showing a markedly shrunken and damaged appearance.

*Immunophenotyping of rosetting cell populations*

Table II details the immunophenotypes of the allogeneic peripheral blood mononuclear cell populations adherent to L428 and L591. The vast majority of the rosetting population were T-cells, evidenced by the expression of CD3 antigen by 93% and 91% of the L428 and L591 adherent cell population respectively. Of these adherent T-cells, 36% and 27%, for L428 and L591 respectively, were of the T-suppressor/cytotoxic (CD8+) subset and 33% and 28%, respectively, of the T-helper/inducer (CD4+) subset. Only a small minority of the peripheral blood cells adherent to L428 and L591 were B-cells or monocytes as shown by the expression of CD37 or CD14 antigens (Table II).

*Adherence of fractionated mononuclear cell populations to Hodgkin's cell lines*

Table III details the degree of enrichment obtained for CD3+, CD4+ and CD8+ T-cells and CD37+ B-cells

Figure 3 Histograms showing the effects of (a) temperature (4–37°C) and (b) $10^{-2}$ M EDTA at pH 7.2 amd 4.5, on the mean number of adherent lymphocytes □ and the percentage of rosettes forming Hodgkin's cells ▨. Bars represent one standard deviation.

Figure 4 The effects of pH (2–10) on the mean number of lymphocytes forming rosettes with L428 or L591 ● and on the percentage of Hodgkin tumour cells forming rosettes ○.

obtained following negative selection by panning normal peripheral blood lymphocytes with the various described monoclonal antibodies. Enrichment for T-cells was very effective, 82% purity being achieved with only 6% contaminating B-cells and no detectable monocytes. Enrichment for CD4+ cells from the CD3+ enriched population was also good, with 63% expressing CD4 and with only 6% contaminating CD8+ cells and 2% CD37+ B-cells. Enrichment for CD8+ cells from the CD3+ enriched population was less satisfactory, 59% of the cells expressing CD8 antigen but with 26% contaminating CD4+ cells and

**Table II** Immunophenotypes of peripheral blood mononuclear cell populations forming rosettes with the Hodgkin cell lines L428 and L591

| *Mab* | *CD* | *Phenotype* | *No. positive rosetting cells/ total no. rosetting cells (%)* L428 | L591 |
|---|---|---|---|---|
| UCHT1[a] | CD3 | T-cell | 119/139 (93%) | 49/54 (91%) |
| OKT8[b] | CD8 | $T_s$-cell | 52/146 (36%) | 17/64 (27%) |
| OKT4[b] | CD4 | $T_h$-cell | 48/147 (33%) | 19/68 (28%) |
| WR17[c] | CD37 | B-cell | 6/114 (5%) | 2/81 (2%) |
| UCHM1[d] | CD14 | MØ | 3/125 (2%) | 7/79 (9%) |

[a]Beverley & Callard (1981); [b]Engleman *et al.* (1981); [c]Moore *et al.* (1987); [d]Hogg, unpublished.

**Table III** Expression of T-cell, B-cell and monocyte markers by normal peripheral blood mononuclear cells enriched for T-cells, $T_h$-cells, $T_s$-cells, B-cells and monocytes by negative selection on panning

| *Enriched population* | *% enriched cell population expressing* UCHT1 (CD3) | OKT4 (CD4) | OKT8 (CD8) | UCHM1 (CD14) | WR17 (CD37) |
|---|---|---|---|---|---|
| Unfractionated PB mononuclear cells | 36 | 15 | 21 | 23 | 12 |
| T-cell[a] (CD3+) | 82 | 34 | 19 | 0 | 6 |
| $T_h$-cell[b] (CD4+) | 78 | 63 | 6 | 0 | 2 |
| $T_s$-cell[c] (CD8+) | 91 | 26 | 59 | 1 | 8 |
| B-cell[d] (CD37+) | 28 | 16 | 5 | 0 | 64 |
| MØ[e] (CD14+) | 5 | 0 | 0 | 0 | 93 |

Populations were negatively selected by depletion of cell populations with the following monoclonal antibodies: [a]UCHM1 (CD14), WR17 (CD37) T-cell enriched; [b]OKT8 (CD8) depletion of T-cell enriched population; [c]OKT4 (CD4) depletion of T-cell enriched population; [d]UCHT1 (CD3), UCHM1 (CD14) enriched for B-cells; [e]Enriched monocytes obtained from Percoll (s.g. 1.06) top fraction.

8% CD37+ B-cells. Of the B-cell enriched population, 64% expressed the pan B-cell marker CD37 but 28% of the cells were contaminating CD3+ T-cells. The highest degree of enrichment was obtained for monocytes, comprising 93% cells expressing CD14 antigen with only 5% contaminating T-cells.

The results obtained following rosetting of the fractionated lymphocyte subpopulations and monocytes with L428 and L591 at a ratio of 1:10 are shown in Figure 5. Clearly revealed here is the observation that all T-cells (CD3+, CD4+ and CD8+) and monocytes (CD14+) are equally good at forming rosettes with both Hodgkin's cell lines. Immunophenotypic analysis of the adherent T-cell populations revealed that the majority of the adherent cells were of the relevant enriched cell population. B-cells (CD37+) were poor at forming adhesive associations with either of the Hodgkin's cell lines and only low percentages of the CD37 enriched rosetting populations were actually B-cells, the majority being comprised of contaminating T-cells.

### *Effects of various agents on rosetting*

Figure 6 details the effects of treating the Hodgkin's cell lines L428 and L591 with various chemical agents/enzymes on their ability to form rosettes with allogeneic lymphocytes at a ratio of 1:10. Treatment of L428 and L591 with neuraminidase resulted in a highly significant ($P<0.01$) reduction in the mean number of lymphocytes adherent to both cell lines. There was also a reduction in the observed percentage of tumour cells forming rosettes for both cell lines. A smaller, though only marginally significant ($P<0.05$) reduction in the mean number of adherent lymphocytes was noted with Cytochalasin B for L428 only. Removal of trypsin-sensitive membrane surface proteins from L428 and

**Figure 5** Histograms showing the mean number of fractionated peripheral blood cell populations rosetting with either L428 or L591 □ and the percentage of Hodgkin cells forming rosettes with the same fractionated population ▨. Bars indicate one standard deviation.

**Figure 6** The effects of various chemical agents and enzymes on the mean numbers of lymphocytes rosetting with L428 and L591 □ and the percentage of Hodgkin cells forming rosettes ▨. Bars indicate one standard deviation.

L591 did not result in a significant reduction in the number of adherent lymphocytes, though there was a moderate reduction in the percentage of Hodgkin's cells forming rosettes, which fell to 65% and 45%, respectively (Figure 6). Treatment with EDTA (pH 7.2), colchicine, sodium azide, tunicamycin or phorbol myristate acetate (PMA) had no significant effect on rosetting for either L428 or L591.

*Effects of EDTA on rosetting*

The effects of EDTA ($10^{-2}$ M) on rosetting were further investigated and the results are shown in Figure 3b. In these experiments EDTA was added to Hanks balanced salts solution (HBSS) yielding a solution of pH 4.5. The pH of a portion of the HBSS was adjusted to pH 7.2 by the dropwise addition of 1 M TRIS and rosetting carried out in HBSS at both pH values. Controls consisted of rosettes formed in non-supplemented HBSS (pH 7.2). It is clear from Figure 3b that EDTA at pH 7.2 had only a marginal effect on rosetting, with the mean number of adherent lymphocytes falling from 6.04 to 5.00 for L428 and from 3.97 to 3.64 for L591. When rosetting was carried out in HBSS at pH 4.5, there was a marked, though non-significant, reduction in the mean number of adherent lymphocytes from 6.04 to 3.60 for L428 and a significant reduction for L591 ($P<0.05$) from 3.97 to 1.74. There was also a marked reduction in the percentage of tumour cells forming rosettes in the presence of EDTA at pH 4.5 from 91% to 42% for L428 and from 71% to 51% for L591. This was not observed for EDTA used at pH 7.2.

*The effects of monosaccharide sugars*

The effects of various monosaccharide sugars (100 mM) on rosette formation by L428 and L591 are shown in Table IV. None of these sugars had any significant effect on rosetting for either cell line.

*Cell surface charge (zeta potential)*

Figure 7 shows the scattergraph plots of the measured zeta potentials obtained for the two rosette-forming Hodgkin's lines L428 and L591 and for the non-rosetting Hodgkin's line Co, before and following treatment with neuraminidase. Also shown are zeta potentials obtained for normal peripheral blood B-cells (CD37+), T-cells (CD3+) and monocytes (CD14+). All of the cell types studied migrated towards the cathode, indicating that all cells were negatively charged. Of the Hodgkin's lines, L428 possessed the least surface charge (mean 8.113 mV) which, following neuraminidase treatment, fell significantly ($P<0.001$) to 2.391 with over half the cells showing a total loss of surface charge. L591 and Co possessed similar mean charges of 12.09 and 12.79, respectively, these values falling to 8.54 mV for L591 and 10.87 mV for Co following neuraminidase treatment. The mean zeta potential values for the variety of peripheral blood mononuclear cells studied were all very similar, being 12.16, 12.35 and 12.57 mV for T-cells (CD3+), B-cells (CD37+) and monocytes, respectively. A point worthy of remark here concerns the range of zeta potentials observed for T-cells, which was considerably narrower than observed for either B-cells or monocytes.

*Effects of anti-L428 Fab on lymphocyte adherence*

Table V shows the results obtained when the Hodgkin's cell line L428 was treated with univalent Fab fragments prepared from the IgG fraction of a rabbit anti-L428 antiserum. It was demonstrated immunocytochemically that both the native antiserum and Fab fragments prepared from this specifically bound to L428 cells on cytocentrifuge preparations. Preincubation of L428 cells for 30 min at 4°C with neat anti-L428 Fab produced a significant ($P<0.05$) reduction in the number of adherent lymphocytes from a mean of 4.83 in the control down to 2.50 in the treated cells and a small reduction in the percentage of L428 cells forming rosettes, from 66% to 57%.

### Discussion

These studies show clearly that the major requirements for the attachment of allogeneic peripheral blood lymphocytes to the Hodgkin's cell lines L428 and L591 are the same. This would probably indicate that the cell adhesion mechanism is also the same for the two cell lines, though clearly L428 is capable of binding a greater number of peripheral blood lymphocytes than L591. This may be accounted for by the fact that an average L428 cell is considerably larger (20–80 μm) than an average L591 cell (10–20 μm) and is

**Table IV** Effects of various monosaccharide sugars (100 mM) on rosetting by L428 and L591

| | *Mean no. rosetting lymphocytes/ Hodgkin cell ± s.d.* | | *% Hodgkin cells forming rosettes* | |
|---|---|---|---|---|
| | *L428* | *L591* | *L428* | *L591* |
| Control | 5.61 ± 1.73 | 4.12 ± 2.61 | 79 | 62 |
| β-D-glucose | 6.19 ± 2.78 | 4.64 ± 1.83 | 84 | 54 |
| D-(+) galactose | 5.19 ± 2.84 | 4.97 ± 2.46 | 76 | 69 |
| L-(−) fucose | 6.21 ± 1.06 | 3.81 ± 2.77 | 79 | 66 |
| Methyl-D-mannoside | 5.50 ± 2.39 | 4.00 ± 1.74 | 86 | 66 |
| N-acetyle-D-glucosamine | 5.86 ± 1.84 | 4.89 ± 1.17 | 81 | 71 |
| N-acetyl neuraminic acid (pH 7.2) | 6.17 ± 1.84 | 3.06 ± 1.19 | 84 | 56 |

**Figure 7** Scattergraph of measured zeta potentials (mV) for the two rosetting Hodgkin's cell lines L428 and L591, and the non-rosetting Hodgkin line. Co, before and after treatment with neuraminidase. Measured zeta potentials for T-cells, B-cells and monocytes are also shown. Bars indicate one standard deviation either side of the calculated mean ($\bar{x}$) shown numerically at the top of each column.

therefore physically capable of binding a larger rosetting population. Both cell lines were derived from different patients with the nodular sclerosing (NS) subtype of Hodgkin's disease (Diehl *et al.*, 1982). On the basis of immunophenotypic and genotypic evidence L428 would appear to be derived from an activated pre-B-cell (Falk *et al.*, 1987) while L591 is positive for Epstein–Barr nuclear antigen (Diehl *et al.*, 1982), indicating a probable derivation from an Epstein–Barr virus (EBV) immortalised lymphocyte from within the Hodgkin's lesion (Weiss *et al.*, 1987) and it is therefore unlikely to be related to the Hodgkin tumour cell population. The major binding requirements for allogeneic peripheral blood lymphocytes to L428 and L591 compared with those for freshly isolated RS/HM cells with autologous intratumoral-derived lymphocytes, as reported by Payne *et al.* (1980), are shown in Table VI. There are clearly major differences between the two systems, namely the temperature independence, lack of requirement for divalent cations and lack of requirement for trypsin sensitive surface membrane proteins by the cell line system, all of which are absolute requirements for the fresh RS/HM cell system (Payne *et al.*, 1980). This might be immediately seen to indicate that the adhesion mechanism for the HD cell lines and for freshly isolated RS/HM cells is different and therefore an unrelated biological phenomenon. However, before drawing such a conclusion certain key differences between the cell line and fresh RS/HM cell system must be taken into consideration. In the first instance, the cell line rosetting system uses allogeneic rosetting populations, unlike the fresh tissue system which is completely autologous, employing rosetting lymphocytes intrinsic to the tumour. The Hodgkin's tumour-derived autologous lymphocyte populations may also possess properties determined by their state of activation, and this may in turn modify their adherence characteristics. Secondly, there are important differences between the cell line and fresh RS/HM cell systems in terms of the way in which the inhibitory effects of the various agents on rosetting are studied. To investigate the effects of an agent in Payne's fresh RS/HM system, existing preformed rosettes in suspension were exposed to a particular agent, and thus the Hodgkin's tumour cell and adherent population(s) were exposed to the agent. This is quite different from the cell line system in the present study, where the HD cell line alone was exposed to the agent under study. While it can be argued that these fundamental differences might account for the observed requirement differences between the cell line and fresh tissue cell suspension systems as listed in Table VI, this seems unlikely in the view of the present authors. The differences are so fundamental as to suggest that completely different adhesion mechanisms exist for the two.

It is clear from out studies that divalent cations are not a requirement for cell line rosetting, an observation which we have made on numerous separate occasions. Where chelating $Mg^{2+}$ and $Ca^{2+}$ with EDTA did result in a decrease in rosetting in the present study, this was clearly shown to be a pH-related effect. These findings contrast sharply with those of Payne *et al.* (1980), where divalent cations were an absolute requirement for rosette formation and maintenance. Removal of membrane surface sialic acid from both Hodgkin's cell lines with neuraminidase produced an almost total abrogation of lymphocyte adherence, again in contrast to the observations of Payne *et al.* (1980), who observed that neuraminidase treatment has no appreciable effect on rosetting. The obvious conclusion to be drawn here is that terminal sialic acid residues on cell surface glycoconjugates expressed by the HD cell lines form an essential element in lymphocyte adhesion, perhaps constituting part of a receptor structure. Treatment of L428 or L591 with the *N*-glycosylation inhibitory agent tunicamycin did not, however,

**Table V** Effects of treatment of L428 cells with Fab' prepared from rabbit anti-L428 hyperimmune antisera upon their rosette forming ability

| *Titre anti-L428 Fab'* | *Mean no. rosetting lymphocytes/ L428 cell ± s.d.* | *% L428 cells forming rosettes* |
|---|---|---|
| – | 4.83 ± 2.192 | 66% |
| Neat (1 mg ml$^{-1}$) | 2.50 ± 1.395[a] | 57% |
| 1:10 | 5.00 ± 3.427 | 79% |
| 1:100 | 4.10 ± 1.523 | 82% |
| 1:1000 | 5.00 ± 2.788 | 73% |

[a]$P < 0.005$.

**Table VI** Some major requirements for rosette formation between lymphocytes and freshly isolated Hodgkin tumour cells and Hodgkin's-derived cell lines

| | *Temp* | *pH* | *Active metabolism* | *Divalent cations* | *Micro tubules* | *Sialic acid* |
|---|---|---|---|---|---|---|
| Fresh HD tissue rosettes (Payne *et al.* 1980) | | ? | – | + | + | – |
| Hodgkin cell line rosettes | – | + | – | – | +(?) | + |

inhibit lymphocyte adherence, indicating that glycoproteins with *N*-linked carbohydrate are unlikely to be involved. This however, does not exclude the involvement of *O*-glycoside linked glycoproteins, glycolipids or proteoglycans. The significant inhibition of lymphocyte adherence to L428 by high concentrations ($1\,mg\,ml^{-1}$) of Fab fragments prepared from a rabbit anti-L428 antiserum would probably indicate that this antiserum contains antibodies directed against a structure(s) involved in adherence. This activity, however, rapidly declined at levels below $1\,mg\,ml^{-1}$, suggesting that only very low levels of specific antibody were present. We are currently investigating the possibility of using such an antiserum for identifying the relevant adhesion structure(s) expressed by the L428 cell.

An alternative possiblity is that adhesion may result from electrical charge differences between the Hodgkin's cell lines and lymphocytes, but it is difficult to equate this possibility with the observations made in the present study. Both L428 and L591 carry net negative electrical charges, as do T-cells, B-cells and monocytes, and all should therefore predictably repel each other. It is perhaps worth noting that L428, the best rosetting cell line, possessed the smallest surface charge of all cells studied and, moreover, this charge disappeared completely in over half of the cells studied, following removal of cell surface sialic acid with neuraminidase, a treatment which concomitantly abolishes their rosetting capability. L591 does not rosette as efficiency as L428 and possesses a mean surface charge comparable with lymphocytes and monocytes. These data make it seem unlikely that Hodgkin cell surface charge is involved in lymphocyte adherence. The coincidental loss of rosetting capability with decrease and even total loss of cell surface charge in the case of L428 following neuraminidase treatment points to an important involvement of sialic acid in lymphocyte adherence. However, it was not possible to inhibit lymphocyte adherence to either L428 or L591 with sialic acid in solution, possibly indicating that this monosaccharide sugar comprises only part of the receptor structure involved in adherence. One observation favouring electrical charge-mediated adherence is that of the occasional formation of multiple layers of lymphocytes sometimes seen to surround L428. These are probably not artefacts of cytocentrifugation, as similar multilayer formations have been observed by us following the culturing of L428 and lymphocytes together in flat bottomed wells. However, when such multilayered rosettes do occur, they appear to be associated with filaments emanating from the L428 cell (Figure 1d) and in these instances lymphocytes appear to be entrapped or adherent to these structures. Similar filaments have been observed for freshly isolated RS cells by Stuart *et al.* (1977) who termed these 'porcupine quills'.

Immunophenotypic analysis demonstrates that unfractionated peripheral blood cell populations forming associations with L428 and L591 are predominantly CD3+ T-cells of which there are approximately equal numbers of adherent CD4+ and CD8+ cells, there being a slight excess of CD4+ cells for L591. Only very few B-cells (CD37+) or monocytes (CD14+) were shown to adhere. The ratio of CD4+ to CD8+ T-cell subsets in peripheral blood is approximately 65:35 (Reinhertz *et al.*, 1979, 1980), and the observations that equal numbers of CD4+ and CD8+ T-cells from the unfractionated peripheral blood mononuclear cell fraction adhere to the Hodgkin's cell line indicates that the CD4+ subset forms associations less efficiently.

B-cell enriched fractions were generally poor at adhering to both L428 and L591 but peripheral blood monocytes were found to be at least as good as T-cells. We must therefore assume that, whatever the mechanism of attachment might be, T-cells, a proportion of B-cells and all monocytes adhere to the Hodgkin cell lines through a common mechanism(s). Morris & Stuart (1984) found that CD4+ T-cells attached more frequently to RS/HM cells than CD8+ T-cells in fresh cell suspensions prepared from Hodgkin's involved lymph nodes. Previous studies have shown that for the majority of HD cases of all subtypes studied, with the exception of the lymphocyte depleted subtype, the majority of the T-cell population in the involved node are of the CD4+ subset (Dorreen *et al.*, 1982; Poppema *et al.*, 1982; Abdulaziz *et al.*, 1984) and thus, the apparently more frequent association of CD4+ T-cells with RS/HM cells may simply reflect the higher proportion of CD4+ cells available to compete for attachment.

We feel it likely that lymphocyte/monocyte adherence to L428 and L591 is mediated by the same or very similar mechanisms, but feel it unlikely, despite certain similarities, that this phenomenon is related to the rosetting reaction observed between RS/HM cells and T-lymphocytes in Hodgkin's-involved tissue *in situ*. Several leucocyte adherence molecules have been described in recent years: LFA antigens (CD11/CD18) mediate leucocyte adherence responses (Pattarroyo *et al.*, 1987; Suomalainen *et al.*, 1986; Kishimoto *et al.*, 1987); CD2 antigen expressed by thymocytes binds to its natural ligand LFA-3 expressed on thymic epithelial cells (Singer *et al.*, 1987).

Recently, Sanders *et al.* (1988) convincingly demonstrated that the adherence of peripheral blood T-lymphocytes and a T-cell clone (8.2) to the HD cell line L428 was mediated via two distinct molecular adhesion systems: LFA-3/CD2 and LFA-1/ICAM-1, with the former system playing the more dominant role. Thus, our observations that B-cells form only inefficient associations with L428 may be accounted for by their lack of surface CD2 expression, thus disabling the major LFA-3/CD2 pathway but leaving the less efficient LFA-1/ICAM-1 system intact, as LFA-1 (CD11a) is expressed by B-cell subsets and ICAM-1 by L428. However, this does not explain why peripheral blood monocytes which express LFA-1, but like B-cells do not express CD2, are equally as good as T-cells at binding to the HD cell lines. There may thus be an alternative system aiding monocyte adherence to the HD cell lines. It has recently been shown that CD4 and class II MHC molecules interact to mediate cell adhesion (Doyle & Strominger, 1987). Peripheral blood monocytes do express small amounts of surface CD4 and L428 and L591 similarly express class II molecules on their surface, albeit variably. It is therefore conceivable that this adhesion pathway is operating in monocyte-L428/L591 interactions, perhaps in an additive fashion with the LFA-1/ICAM-1 system. That the CD4/class II system is operative in the HD cell line rosette system is supported by our own observations that T-cell–L428 rosette formation is partially, though non-significantly, inhibited by antibodies to monomorphic determinants of the alpha chain of the HLA DR molecule. We have not yet investigated this using monocytes as the rosetting population.

This work was generously supported by a grant from the Cancer Research Campaign. We are grateful to Dr Eamonn Rooney for undertaking cell electrophoretic measurements.

### References

ABDULAZIZ, Z., MASON, D.Y., STEIN, H., GATTER, K.C. & NASH, J.R.G. (1984). An immunohistological study of the cellular constituents of Hodgkin's disease using a monoclonal antibody panel. *Histopathology*, **8**, 1.

ARCHIBALD, R.B. & FRENSTER, J.H. (1973). Quantitative ultrastructural analysis of in vivo lymphocyte/Reed–Sternberg cell interactions in Hodgkin's disease. *Natl Cancer Inst. Monograph*, **36**, 239.

BEVERLEY, P.C.L. & CALLARD, R.E. (1981). Distinctive functional characteristics of human 'T' lymphocytes defined by E rosetting on a monoclonal anti-T-cell antibody. *Eur. J. Immunol.*, **11**, 329.

DIEHL, V., KIRCHNER, H.H., BURRICHTER, H. & 9 others (1982). Characteristics of Hodgkin's disease-derived cell lines. *Cancer Treat. Rep.*, **66**, 615.

DORREEN,. M.S., HABESHAW, J.A., WRIGLEY, P.F.M. & LISTER, T.A. (1982). Distribution of T-lymphocyte subsets in Hodgkin's disease characterised by monoclonal antibodies. *Br. J. Cancer*, **45**, 491.

DOYLE, C. & STROMINGER, J.L. (1987). Interaction between CD4 and Class II MHC molecules mediates cell adhesion. *Nature*, **330**, 256.

EISENBERG, M., GRESALFI, T., RICCIO, T. & McLAUGHLIN, S. (1979). Adsorption of monovalent cations to bilayer membranes containing negative phospholipids. *Biochemistry*, **18**, 5213.

ENGLEMAN, E.G., BENIKE, C.J., GLICKMAN, E. & EVANS, R.L. (1981). Antibodies to membrane structures that distinguish supressor/cytotoxic and helper T-lymphocyte subpopulations block the mixed leucocyte reaction in man. *J. Exp. Med.*, **154**, 193.

FALK, M.H., TESCH, H., STEIN, H. & 4 others (1987). Phenotype versus immunoglobulin and T-cell receptor genotype of Hodgkin's-derived cell lines: Activation of immature lymphoid cells in Hodgkin's disease. *Int. J. Cancer*, **40**, 262.

HENNEY, C.S. & BUBBERS, J.E. (1973). Antigen-T-lymphocyte interactions: Inhibition by cytochalasin. *Br J. Immunol.*, **3**, 85.

JONDAL, M., KLEIN, E. & P YEFENOF, E. (1975). Surface markers on human T & B-lymphocytes. VII. Rosette formation between peripheral T-lymphocytes and lymphoblastoid cell lines. *Scand. J. Immunol.*, **4**, 259.

JONES, D.B., SCOTT, C.S., WRIGHT, D.H. & 4 others (1985). Phenotypic analysis of an established cell line derived from a patient with Hodgkin's disease. *Hematol. Oncol.*, **3**, 133.

KADIN, M.E., NEWCOM, S.R., GOLD, S.B. & STITES, D.P. (1974). Origin of the Hodgkin's cell. *Lancet*, **ii**, 167.

KAY, M.M.B. (1976). Hodgkin's disease: A war between T-lymphocytes and transformed macrophages. *Rec. Results Cancer Res.*, **56**, 111.

KISHIMOTO, T.K., MILLER, L.J. & SPRINGER, T.A. (1987). Homology of LFA-1, Mac-1 and P150,95 with the extracellular matrix receptors defines a novel supergene family of adhesion proteins. In *Leucocyte Typing III*, McMichael, A.J. (ed) p. 896. Oxford University Press: Oxford.

LAEMMLI, U.K. (1970). Cleavage of structural proteins during the assembly of the head of bacteriophage T4. *Nature*, **227**, 680.

LEE, A.G., EAST, J.M., JONES, O.T., McWHIRTER, J., ROONEY, E.K. & SIMMONDS, A.C. (1982). Interaction of fatty acids with the calcium-magnesium ion dependent adenosinetriphosphatase from sarcoplasmic reticulum. *Biochemistry*, **21**, 6441.

LIPSKY, P.E. & ROSENTHAL, A.S. (1973). Macrophage-lymphocyte interaction. I. Characteristics of the antigen-independent binding of guinea pig thymocytes and lymphocytes to syngeneic macrophages. *J. Exp. Med.*, **138**, 900.

MOORE, K., COOPER, S.A. & JONES, D.B. (1987). Use of the monoclonal antibody WR17 identifying the CD37 gp40–45 Kd antigen complex in the diagnosis of B-lymphoid malignancy. *J. Pathol.*, **152**, 13.

MORRIS, C.S. & STUART, A.E. (1984). Reed–Sternberg/lymphocyte subpopulations as defined by monoclonal antibodies. *J. Clin. Pathol.*, **37**, 767.

PATARROYO, M. & ANSOTEGUI, I.J. (1987). Effect of monoclonal antibodies of non-lineage CD11 and CD18 panels on phorbol ester-induced adhesion among different leucocytes. In *Leucocyte Typing III*, McMichael, A.J. (ed) p. 839. Oxford University Press: Oxford.

PAYNE, S.V., JONES, D.B. & WRIGHT, D.H. (1977). Reed–Sternberg cell/lymphocyte interaction. *Lancet*, **ii**, 768.

PAYNE, S.V., NEWELL, D.G., JONES, D.B. & WRIGHT, D.H. (1980). The Reed–Sternberg cell/lymphocyte interaction. Ultrastructure and characteristics of binding. *Am. J. Pathol.*, **100**, 7.

POPPEMA, S., BHAN, A.K., REINHERZ, E.L., POSNER. M.R. & SCHLOSSMAN, S.F. (1982). *In situ* immunologic characterisation of cellular constituents in lymph nodes and spleens involved by Hodgkin's disease. *Blood*, **59**, 226.

REINHERTZ, E.L., KUNG, P.C., GOLDSTEIN, G., LEVEY, R.H. & SCHLOSSMAN, S.F. (1980). Discrete stages of human intrathymic differentiation: Analysis of normal thymocytes and leukemic lymphoblasts of T-cell lineage. *Proc. Natl Acad. Sci. USA*, **77**, 1588.

REINHERTZ, E.L., KUNG, P.C., GOLDSTEIN, G. & SCHLOSSMAN, S.F. (1979). Further characterisation of the human inducer T-cell subset defined by a monoclonal antibody. *J. Immunol.*, **123**. 2894.

ROONEY, E.K. & LEE. A.G. (1983). Binding of hydrophobic drugs to lipid bilayers and to the ($Ca2+ + Mg2+$) ATPase. *Biochim. Biophys. Acta*, **732**, 428.

SANDERS, M.E., MAKAGOBA, M.W., SUSSMAN, E.H., LUCE, G.E.G., COSSMAN, J. & SHAW, S. (1988). Molecular pathways of adhesion in spontaneous rosetting of T-lymphocytes to the Hodgkin's cell line L428. *Cancer Res.*, **48**, 37.

SCHAADT, M., DIEHL, V., STEIN, H., FONATSCH, C. & KIRCHNER, H.H. (1980). Two neoplastic cell lines with unique features derived from Hodgkin's disease. *Int. J. Cancer*, **26**, 723.

SINGER, K.H., WOLD, L.S., SPRINGER, T.A., DENNING, S.M., TUCK, D.T. & HAYNES, B.F. (1987). Human thymocyte binding to autologous and allogeneic thymic epithelial cells is inhibited by CD2 and LFA-3 monoclonal antibodies. In *Leucocyte Typing III*, McMichael, A.J. (ed) p. 140. Oxford University Press: Oxford.

STUART, A.E., WILLIAMS, A.R.W. & HABESHAW, J.A. (1977). Rosetting and other reactions of the Reed–Sternberg cell. *J. Pathol.*, **122**, 81.

SUNDEEN, J., LIPFORD, E., UPPENKAMP, M. & 4 others (1987). Rearranged antigen receptor genes in Hodgkin's disease. *Blood*, **70**, 96.

SUOAMALAINEN, H., GAHMBERG, C.G., PATARROYO, M., BEATTY, P.G. & SCHRODER, T. (1986). Genetic assignment of gp90, leucocyte adhesion glycoprotein to human chromosome 21. *Somat. Cell Mol. Genet.*, **12**. 297.

VAN PARYS, G., VAN DEN OORD, J., DE WOOLF-PEETERS, C., DE VOS, R. & DESMET, V.J. (1985). Reed–Sternberg cells, lymphocytes and interdigitating reticulum cell rosettes in Hodgkin's disease. *J. Clin. Pathol.*, **38**, 1316.

WEISS, L.M., STRICKLER, J.G., WARNKE. R.A., PURTILO, D.T. & SKLAR, J. (1987). Epstein-Barr viral DNA in tissues of Hodgkin's disease. *Am. J. Pathol.*, **129**, 86.